# Next Generation Sequencing for Gene Fusion Analysis in Lung Cancer: A Literature Review

**DOI:** 10.3390/diagnostics10080521

**Published:** 2020-07-27

**Authors:** Rossella Bruno, Gabriella Fontanini

**Affiliations:** 1 Unit of Pathological Anatomy, University Hospital of Pisa, Via Roma 67, 56126 Pisa, Italy; rossella.bruno@for.unipi.it; 2 Department of Surgical, Medical, Molecular Pathology and Critical Area, University of Pisa, Via Savi 10, 56126 Pisa, Italy

**Keywords:** next generation sequencing, gene fusions, lung cancer, solid and liquid biopsy

## Abstract

Gene fusions have a pivotal role in non-small cell lung cancer (NSCLC) precision medicine. Several techniques can be used, from fluorescence in situ hybridization and immunohistochemistry to next generation sequencing (NGS). Although several NGS panels are available, gene fusion testing presents more technical challenges than other variants. This is a PubMed-based narrative review aiming to summarize NGS approaches for gene fusion analysis and their performance on NSCLC clinical samples. The analysis can be performed at DNA or RNA levels, using different target enrichment (hybrid-capture or amplicon-based) and sequencing chemistries, with both custom and commercially available panels. DNA sequencing evaluates different alteration types simultaneously, but large introns and repetitive sequences can impact on the performance and it does not discriminate between expressed and unexpressed gene fusions. RNA-based targeted approach analyses and quantifies directly fusion transcripts and is more accurate than DNA panels on tumor tissue, but it can be limited by RNA quality and quantity. On liquid biopsy, satisfying data have been published on circulating tumor DNA hybrid-capture panels. There is not a perfect method for gene fusion analysis, but NGS approaches, though still needing a complete standardization and optimization, present several advantages for the clinical practice.

## 1. Introduction

Fusion genes are hybrid genes generated by the juxtaposition of two previously independent genes, following structural rearrangements like deletions, inversions, translocations or duplications within the same chromosome or between different chromosomes [1]. Currently, more than 10,000 gene fusions have been identified in human cancers, many of which are strong driver alterations [1]. Precision medicine in non-small cell lung cancer (NSCLC) and particularly in metastatic advanced lung adenocarcinoma (ADC), is evolving rapidly with an increased number of targetable alterations [2,3]. Consequently, molecular testing guidelines are continuously updated on the basis of available clinical data, taking into account genetic discovery, new drug approval and new molecular techniques [2]. According to the recent updated guidelines, molecular testing in NSCLC includes three classes of targets: the “must-test” biomarkers with approved targeted therapies (i.e., epidermal growth factor receptor (*EGFR)*); “should-test” biomarkers (i.e., MET proto-oncogene, receptor tyrosine kinase (*MET*)), crucial to eventually direct patients to ongoing clinical trials and “investigational” biomarkers, without a clear clinical value at the moment. Molecular characterization has then to be performed using highly sensitive, specific, adequate and validated tests allowing the analysis of samples with a low percentage of tumor cells (10–20%) and working both on histological and cytological specimens. Overall, whenever possible, the use of multigene testing panels, particularly next generation sequencing (NGS) ones, ensures a wider definition of the molecular landscape of lung tumors including all biomarker types, common and rare [2].

Among targetable alterations with approved drugs in ADC there are different fusion proteins leading to constitutively activated kinases. In detail, patients with ADC harboring anaplastic lymphoma kinase (*ALK*), ROS proto-oncogene 1 (*ROS1*), rearranged during transfection proto-oncogene gene (*RET*) and neurotrophin kinase (*NTRK*) gene rearrangements can benefit from a treatment with tyrosine kinase inhibitors (TKIs), so an efficient and valued molecular testing is mandatory [2,3].

*ALK* rearrangements have a frequency of 2–7% among advanced lung ADC patients and five ALK inhibitors are currently available: the first generation crizotinib, the second generation alectinib, ceritinib and brigatinib and the third generation lorlatinib [4]. Different clinical trials demonstrated a better efficacy for the second generation drugs alectinib and brigatinib in comparison to crizotinib in the first line treatment of *ALK*-rearranged NSCLC [3]. The third generation lorlatinib, which is the only TKI able to target the *ALK* resistance mutation G1202R, has been approved both as a second and as a third line treatment after other ALK first or second generation TKIs [3]. The *ALK* gene is located on chromosome 2p23.2 and several fusion partners have been described, with the most common being the echinoderm microtubule-associated protein like-4 (*EML4*) gene located on the same chromosome (2p21). The breakpoint site can occur in different exons, consequently forming various fusion variants associated with different TKI sensitivity [5]. *ROS1* rearrangements have a frequency of 1–2% and crizotinib is currently the only approved TKI for the first line treatment [4,6]. However, in recent clinical trials it has been demonstrated that central nervous system activity of other TKIs targeting also ROS1 (ceritinib and entrectinib) is superior to crizotinib [3]. Among the best known *ROS1* fusion partners there are CD74 molecule (*CD74*), solute carrier family 34 member 2 (*SLC34A2*) and Golgi-associated PDZ and coiled-coil motif containing (*GOPC*) [7]. *NTRK* fusions approximatively occur in 0.1 to 1% of NSCLC cases and the *NTRK* genes are *NTRK1*, *NTRK2*, and *NTRK3*, located on chromosomes 1q23.1, 9q21.33 and 15q25.3 respectively. They encode for tropomyosin receptor tyrosine kinases TRKA (*NTRK1* gene), TRKB (*NTRK2* gene), and TRKC (*NTRK3* gene) functioning during normal neuronal development and maintenance [8]. Tumors harboring *NTRK* fusions can benefit from a treatment with the TKIs larotrectinib and entrectinib [9]. The *RET* gene is located on chromosome 10q1.2 and its rearrangements are detected in 1–2% of ADC with the most common fusion partner represented by the kinesin family member 5B (*KIF5B)*. Over the years, different RET inhibitors have been developed and evaluated for the treatment of *RET*-rearranged tumors, including both multikinase inhibitors, like cabozantinib and vandetanib, and selective inhibitors, like pralsetinib and selpercatinib [4,10,11]. The multikinase inhibitors have shown an unsatisfying clinical efficacy with different off-target adverse events and a low overall response rate. On the other hand, selective inhibitors have proved to be more effective with a better toxicity profile [10]. Recently, selpercatinib has been approved from the Food and Drug Administration (FDA) for the treatment of *RET*-rearranged NSCLC [12].

The development and approval of new drugs targeting also rare gene fusions makes necessary a deep molecular characterization of lung cancer specimens, in order to provide patients with the best therapeutic options [3].

Several techniques are available to evaluate gene fusions, which can be divided in single gene testing and multiplex testing, conventional and next generation methods, DNA, RNA or protein based. Conventional and single test methods include fluorescence in situ hybridization (FISH), immunohistochemistry (IHC) and a retro-transcription polymerase chain reaction (RT-PCR) [13].

FISH detects gene rearrangements at DNA level; it is mainly based on the use of break apart probes and does not require an a priori knowledge of the fusion partner. In many cases it is considered the gold standard and can discriminate rearrangements from polysomy and amplification, but it does not allow a determination of the exact fusion variant. Break apart probes can miss small intrachromosomal rearrangements and not all the identified DNA rearrangements produce an expressed fusion transcript [4,14]. IHC detects fusion proteins, besides the specific fusion variant: whenever an in-frame rearrangement occurs, the 3-prime (3′) region of the oncogene is juxtaposed with the 5-prime (5′) sequence of a fusion partner gene highly expressed in tumor cells, leading to an active and expressed oncoprotein [4,13]. IHC has a good sensitivity, almost all molecular pathology laboratories are familiar with this method, it is time saving and also easily automatable, cost-friendly, and different clinically validated antibodies are available [15]. Treatment decisions can be made when ALK IHC results are clearly positive, whereas positive ROS1 and NTRK IHC results have to be confirmed by orthogonal techniques [2,3]. Although multiplexing has been improved, FISH and IHC usually require a test for each gene to analyze, expert pathologists are needed and results can be influenced by interobserver variation [16]. RT-PCR allows a highly sensitive detection of fusion transcripts at RNA levels, but it requires primer pairs specific for the known fusion to investigate. This approach suffers from RNA quality, which is often poor from formalin-fixed and paraffin-embedded (FFPE) samples, allows the analysis of few variants at time and misses all the unknown variants [8,13].

Unfortunately, most NSCLC patients are diagnosed in advanced stages and available material is often represented by limited biopsies or scarce fine needle aspiration (FNA) specimens, and single gene testing can be a limiting approach [17]. Moreover, the ever-growing list of predictive biomarkers has encouraged and favored the development of multiplex techniques. In this context, it is possible to discriminate NGS and non-NGS methods. Among non-NGS methods the NanoString nCounter System is one of the most valuable for gene fusion analysis [13,16]. It is based on a digital color-coded barcode technology allowing to directly count specific mRNA molecules without any retro-transcription or amplification step, thus being adequate also for the analysis of degraded clinical samples [18]. This system can reach a high sensitivity and specificity, with a good throughput allowing an analysis of up to 800 targets for 12 samples simultaneously. Gene fusion analysis by NanoString depends on a dual strategy: evaluation of the 3′ and 5′ gene region imbalance and detection of known fusion transcripts through the use of target-specific probes. Although most data about gene fusion analysis by NanoString have been obtained on histological specimens, it has been proved that it may be successfully applied also on cytological ones [16]. Moreover, new assays based on the 3D biology (NanoString Technologies) have been recently developed, making it possible to analyze DNA, RNA and protein alterations together, but few data are available about their application on clinical samples [13]. Another RNA–based non-NGS method for the detection of gene fusions uses the iPLEX chemistry and matrix-assisted laser desorption ionization time-of-flight analysis on a MassARRAY mass spectrometry platform. Specific tests to investigate the presence of *ALK*, *ROS1* and *RET* rearrangements in lung cancer have been developed by Agena Bioscence. Some tests are based on screening assays evaluating the presence of imbalanced expression between exons upstream and downstream the kinase domain without characterizing the fusion variant, whereas other available tests specifically investigate known fusion variants and breakpoints. Overall, this approach requires a retro-transcription step, amplification, labeling and detection using mass spectrometry. Both NanoString and Agena systems showed a good concordance with conventional tests and NGS approaches [19].

Finally, in the field of precision medicine, NGS or massively parallel sequencing is really implementing and facilitating tumor molecular characterization besides gene fusion analysis [20]. NGS has not to be considered like a unique entity but it includes several applications and techniques, each with its own strengths and weaknesses.

The aim of this literature review is to summarize NGS approaches specific for gene fusion analysis and their performance on NSCLC clinical samples: histological, cytological specimens and circulating cell-free tumor DNA (ctDNA). Even if different NGS panels are available and have been introduced in the routine procedures of some laboratories, gene fusion testing is still presenting more challenges than other gene variants.

## 2. Data Sources

PubMed was searched using the following keywords and their combinations: next generation sequencing, gene fusions, gene rearrangements, circulating tumor DNA, liquid biopsy and lung cancer. Considering the rapid evolution of NGS techniques, only publications over the last 5 years, written in the English language, were considered. Moreover, only papers specifically concerning the analysis of gene fusions by NGS in NSCLC samples were selected.

This review was divided into the following parts: a brief description of technical principles behind available NGS approaches, methods and platforms, two paragraphs specific for amplicon and hybrid-capture based panels, a part related to gene fusion analysis on liquid biopsy and a general discussion summing up the most relevant aspects.

### 2.1. NGS and Gene Fusion Analysis

NGS approaches for gene fusion analysis can be first divided according to the analysis level: DNA or RNA; then it is possible to discriminate targeted panels (limited numbers of analyzed genes) from the comprehensive whole genome (WGS) or transcriptome (WTS) sequencing. In addition, NGS methods differ for library preparation, target enrichment and sequencing chemistry [9].

Overall DNA-based tests allow the characterization of the exact gene fusion breakpoints together with other genetic alterations (i.e., single nucleotide variants, indels, duplication, copy number variations) and does not require an additional RNA purification step. On the other hand, it does not give indications about expression of the rearranged locus. Moreover, the detection of some fusion events involves intronic regions, which can be extremely large with repetitive sequences (i.e., *NTRK2* and *NTRK3*), thus impairing sequencing efficiency [8].

RNA-based approaches identify only expressed fusion genes and can discriminate splicing isoforms and quantify fusion transcripts. RNA sequencing is not affected by intronic regions. Anyway, to handle RNA is more complicated than DNA, particularly, when purified from FFPE specimens it can be highly degraded and a careful quality check is necessary before starting the NGS analysis [13].

Among comprehensive approaches, WGS is the most global and unbiased one and can identify the genomic location of all known and unknown fusion events. However, it requires a large amount of starting material and mean coverage is usually low. WTS sequencing allows the detection of known and unknown expressed gene fusions, it is more sensitive than WGS, but RNA from tumor clinical samples is barely adequate for this type of analysis in terms of both quality and quantity. In the clinical practice, neither WGS nor WTS are convenient, since these methods are expensive, time consuming and require a complicated data analysis workflow [20].

**Figure 1:**
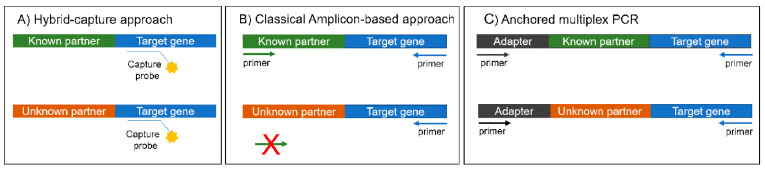
Schematic representation of the main NGS targeted approaches for gene fusion testing. (**A**) Hybrid-capture: gene-specific enrichment by a hybridization step with biotinylated DNA or RNA probes specific for the regions of interest: (**B**) Classical amplicon-based approach: target enrichment by a multiplex PCR using fusion variant specific primers; (**C**) Anchored Multiplex PCR: only one fusion partner needs to be targeted. Briefly, an NGS adapter is ligated to cDNA fragments, target enrichment is based on the amplification between gene-specific primers and a primer to the adapter.

Targeted panels are the most suitable for the clinical practice, require a lower input of starting material, analysis is based on a limited number of clinical valuable targets, are faster and data analysis and result interpretation is easier than with WGS and WTS. DNA- or RNA-targeted panels for gene fusions can be hybrid-capture- or amplicon-based (Table 1). The hybrid-capture method implies a gene-specific enrichment by a hybridization step with probes complementary to the regions of interest, whereas amplicon-based methods rely on a multiplex PCR (mPCR) with primers specific for each target (Figure 1) [20,21,22].

For what concerns NGS platforms, although sharing the same steps, considerable differences are related to the sequencing chemistry. The two most common platforms are based on a sequencing by synthesis: Illumina sequencers (Illumina, San Diego, CA, USA), which use four distinct fluorescently labeled nucleotides and optical imaging to visualize the growing complementary strand; and ThermoFisher sequencers (ThermoFisher Scientific, Waltham, MA, USA) based on the Ion Torrent technology, which uses a non-optical semiconductor-based sequencing with unlabeled nucleotides. Briefly, after library preparation, template molecules are clonally amplified on beads then housed into lots of microwells (each microwell contains only one bead) interfaced to a semiconductor chip. Template DNA on the beads within each well is sequenced by using unlabeled and unmodified nucleotides, which are introduced in the sequencing reaction one at a time in different flows, according to a precise order. Whenever a nucleotide is incorporated, a hydrogen ion is released causing a pH variation, which is detected by the semiconductor chip, chemical signal is then translated into digital signal [23]. Illumina technology permits the use of both a hybridization capture-based method and an amplicon-based approach; Ion Torrent applications only use the amplicon-based method [13]. Other important differences are related to single ends or paired ends sequencing and also to data analysis strategies. Considering gene fusions, most of the available NGS tests use a paired-ends sequencing approach, because it allows an evaluation of both split (chimeric sequence reads encompassing sequence fragments from two different genes, identification of fusion breakpoint) and spanning reads (discordant read pairs: forward read maps on one gene and reverse to a different one) [22]. Finally, analysis procedures can be based on a “mapping first” approach rather than on an “assembly first procedure”. The mapping first approach is the preferred one in many cases: NGS reads are aligned to a reference genome or transcriptome and rearrangements are identified through reads that map to multiple genes. In the assembly first method, overlapping NGS reads are first assembled in longer segments and are then mapped to reference genome or transcriptome to identify discordantly mapping ones. Bioinformatics software and analysis algorithms greatly impact on technical sensitivity and specificity [24]; commercially available panels are usually coupled with specific analysis packages, moreover, different innovative analysis methods for gene fusions are continuously being developed [25,26,27].

### 2.2. Amplicon-Based Approaches

Amplicon-based methods rely on target enrichment by a mPCR; this approach is as ideal for degraded and limited samples as FFPE ones and it can be faster and cheaper than hybrid-capture methods [28]. Amplicon-based methods are not adequate for gene fusion analysis at DNA level because of the presence of large intronic regions often involved in rearrangements which hamper primer design and the optimization of multiplex reactions [22]. Classical mPCR is based on the use of primers flanking exon-exon fusion combinations allowing to detect only known fusion variants (Figure 1). However, RNA-based fusion panels also include testing for expression imbalances between 5′ and 3′ regions of the target genes, thus permitting an identification of the presence of a rearrangement even if the fusion partner is not known and is not included in the panel [21,29]. The amplicon-based approach is one of the most common approaches for gene fusion analysis and different commercial and custom panels have already been tested and validated. Particularly, in most studies, ThermoFisher panels were used on Ion Torrent Platforms (Ion PGM, Ion S5 and Ion Proton). The Ampliseq RNA lung cancer panel, allowing the analysis of about 70 fusions involving *ALK*, *ROS1*, *RET* and *NTRK1* is one of the most diffuse. Overall, with Ampliseq panels, a low RNA input of about 10 ng is required and the successful rate across available studies was satisfying, ranging from 74% [29] to 99% [30]. When compared with FISH, IHC and RT-PCR, this test showed a good concordance, in some cases greater than 90% [28,29,30,31,32,33,34,35,36].

In detail, in one of the largest prospective series, Volckmar and collaborators reported data about a combined targeted DNA (single nucleotide variations, indels, copy number alterations) and RNA (gene fusions) sequencing on the first consecutive 3000 FFPE cases from stage IIIB/IV NSCLC patients. The RNA test was specifically performed on 2003 FFPE cases with a success rate of 93.1%, thus confirming the adequacy of this test for routine specimens [32]. Pfarr’s group evaluated a retrospective and a prospective cohort of NSCLC cases by using the Ampliseq RNA panel. The retrospective series was enriched for gene fusion positive cases (28 *ALK* positive and 7 *ROS1* positive) all confirmed by NGS test. Interestingly, they found 16 out of 28 *ALK* positive cases and only 2 out of 7 *ROS1* positive cases presenting also imbalance between 5′ and 3′ gene regions. This discrepancy between fusion variant calls and imbalance can be due to the presence of gene basal expression, as happens for *ROS1*, and also to expression background from tumor surrounding microenvironment. In the prospective part, 97 out of 109 FFPE NSCLC tested samples were adequate for NGS analysis even if the percentage of tumor cells was as low as 10%. Moreover, they reported an intra and inter-run reproducibility equal to 100% [31]. Vollbrecht and collaborators used the same panel to assess the fusion status of two series of ADC cases, one including 18 cases concordant and one 15 discordant between FISH and IHC. As expected, the RNA NGS test, evaluating only expressed gene fusions, was in greater agreement with IHC than FISH [34].

In another study, Dacic et al. evaluated an RNA panel allowing the analysis of 169 gene fusions in 19 genes, including the imbalance detection of 12 kinases, using an Ion Proton system. Their approach required 20 ng of RNA input and the analysis was performed on 28 *ALK*-positive cases with different FISH signal types: splitted signal, single orange signal or mixed pattern (Vysis ALK Break Apart FISH kit; Abbott Molecular, Illinois, USA). Sixteen positive samples were confirmed by NGS, some of which presented only imbalance, thus suggesting the presence of fusion variants not included in the NGS panel. Notably, discordant NGS cases were mostly in FISH mixed and single orange signal groups; in these two groups IHC was often in agreement with NGS results. They also reported that NGS fusion positive cases were associated with a better response to crizotinib than NGS negative ones [37].

Among RNA amplicon-based methods, a valuable alternative to classical mPCR is the anchored mPCR, which allows the analysis of known and unknown fusion variants, because only one fusion partner needs to be targeted. Briefly, an NGS adapter is ligated to cDNA fragments, then target enrichment is based on the amplification between gene-specific primers and a primer to the adapter (Figure 1). In this way, known and unknown fusions of genes of interest can be detected [38]. The most common anchored mPCR panels are produced by ArcherDX (ArcherDX, Boulder, CO, USA) and have provided excellent results. These panels require a higher RNA input than mPCR assay—about 200 ng [39,40]—and are compatible with both Illumina and ThermoFisher sequencing platforms. Cohen and collaborators evaluated a total of 297 FFPE specimens using the Archer comprehensive Thyroid and Lung Panel with the related analysis software and workflow on an Ion PGM platform. Overall fusion variants had an incidence of 19% with a dropout rate of 18%, equal to 30% on cytological specimens, they included also decalcified samples and many of them failed not only RNA but also DNA testing [39]. Hindi and collaborators, using a custom Archer panel on a NextSeq500 platform (Illumina), analyzed 72 retrospective and 84 prospective FFPE cases (decalcified samples were excluded) and they reported a sensitivity, specificity and reproducibility of 100%; dilution studies confirmed that the limit of detection was equal to 12.5% tumor cells [40]. Other anchor mPCR NGS panels for gene fusion analysis are produced also by Asuragen (Asuragen, Austin, Texas, USA) and Qiagen (Qiagen, Düsseldorf, Germany) [22]. Tachon and collaborators directly compared two of the up-mentioned methods: the FusionPlex^®^ *ALK*, *RET* and *ROS1* v2 Kit (ArcherDX) with the Archer analysis software and the Human Lung Cancer Panel for fusions of *ALK*, *ROS1*, *RET* and 24 other genes (Qiagen) with the specific coupled analysis software, using respectively 50 and 20 ng of total RNA and performing sequencing reactions on an Illumina MiSeq platform. They analyzed 41 FFPE cases already characterized by FISH and IHC. Overall, 4 samples failed both the NGS tests, the Archer panel correctly identified 22 out of 27 positive samples and the Qiagen panel 23, the only discrepancy between the two assays involved one *ALK* rearrangement undetected by the Archer assay; however, this sample did not fulfil the quality assessment of the manufacturer. Three cases found positive by IHC and FISH were found to be negative by NGS, and considering that RT-PCR and Sanger sequencing confirmed the presence of EML4-ALK variants, they are real NGS false negative, mainly due to the low percentage of tumor cells and a low RNA concentration [15]. The two evaluated panels had comparable results for both accuracy and costs and were feasible for molecular testing in routine organizations.

For the concerns of performances of classical mPCR and anchor mPCR, Vendrell and collaborators directly compared it to the Archer Fusion Plex and the Ion AmpliSeq RNA Lung Cancer Research Fusion Panel, because both run on an Ion PGM instrument. They analyzed 37 FFPE NSCLC cases already characterized by FISH and IHC (15 positive and 22 negatives for gene fusions); for the Ampliseq analysis, 10 ng of total RNA was used, whereas for the Archer, 200 ng was used. The Ampliseq panel correctly diagnosed 25 out of 37 cases, with a negative case reported as positive by FISH and IHC and 11 uncertain cases. The Archer panel correctly diagnosed all cases. Interestingly, a fusion variant was called differently by the two NGS approaches: EML4(13)-ALK (19) fusion by Ampiseq and an EML4(13)-ALK(20) fusion by Archer. The fusion was confirmed as EML4(13)-ALK(20) using RT-PCR and Sanger sequencing. Moreover, the Archer panel let to the identification of one novel and two rare *ALK* fusion transcripts: GCC2-ALK (S35), DCTN1-ALK (S36), and CLIP1-ALK (S37); two of these patients were treated and responded to crizotinib. Notably, no sample failed the NGS and the lowest percentage of tumor cells was equal to 20% [41]. Both mPCR and anchor mPCR are valuable methods and comparison data are limited to draw any definitive conclusion. It is clear that anchor mPCR requires a higher amount of RNA input, which in some cases can be a limiting factor, but on the other hand it gives the important advantage to also characterize unknown variants.

Both mPCR and anchor mPCR can suffer from PCR bias, like allele dropout, off-target primers binding and primers dimerization, which can negatively impact on sequencing.

Overall, amplicon-based procedures for gene fusion analysis require RNA purification and imply the use of combined DNA and RNA NGS tests to perform a complete molecular characterization in NSCLC clinical practice. In some cases, it could be difficult to obtain enough material and separate tissue sections. Besides the use of distinct extraction kits for DNA and RNA, both manual or automatized, some groups used a unique kit like the AllPrep FFPE kit (Qiagen) [33] or the RecoverAll (ThermoFisher) [29], allowing them to separate DNA and RNA from the same starting material. Alternative approaches without splitting DNA and RNA have also been proposed. For instance, Haynes and collaborators evaluated a single-workflow amplicon-based targeted NGS panel for NSCLC, covering 135 RNA and 55 DNA disease-relevant targets, using a MiSeq Illumina platform. DNA and RNA were isolated together using a commercial kit—the QIAamp^®^ DNA FFPE Tissue Kit (QIAGEN)—with the exclusion of RNase treatment in order to recover total nucleic acid (TNA), which was evaluated for DNA and RNA yield using specific qPCR assays. Then, DNA and RNA libraries were made following their own protocols and run together. In detail, for RNA libraries, an input of 10 ng TNA was used with the QuantideX^®^ NGS RNA Lung Cancer Kit (Asuragen). They analyzed 109 FFPE core needle biopsies, 110 FFPE surgical resections and 50 FNA smears. For RNA analysis, 94/109 biopsies and all 110 resections were adequate but no FNA smear had sufficient RNA to perform the test. Moreover, their RNA pool enables also the detection of *MET* exon 14 skipping and quantification of 23 other mRNA targets, with a potential prognostic value [42]. In the same way, Song and collaborators developed a single tube dual-template assay together with a specific bioinformatic pipeline. Nucleic acids were purified together without splitting DNA and RNA by the FormaPure FFPE nucleic acid extraction kit (Beckman Coulter, Brea, CA, USA) or the PanoPure FFPE TNA extraction kit (HeliTec Biotechnologies, France) and quantified by specific qPCR assays, then DNA and RNA libraries were constructed in the same tube and sequencing was performed on Illumina platforms. They successfully tested this panel on a cohort of 1000 lung cancer cases; the great advantage is to use in the same assay RNA for gene fusion testing and DNA for single nucleotide variants, indels and copy number variations [43]. In all cases, a careful quantitative and qualitative RNA evaluation is required before retro-transcription to cDNA and sequencing procedures.

### 2.3. Hybrid-Capture Approaches

The hybrid-capture method implies a gene-specific enrichment by a hybridization step with biotinylated DNA or RNA probes specific for the regions of interest (Figure 1). If DNA is analyzed, probes are complementary to intronic, exonic and intergenic regions, whereas if RNA is analyzed, probes target only exonic regions [9,21]. This approach allows to identify known and novel fusion variants for any gene targeted by the capture panel; obviously completely novel fusion genes are omitted, indeed at least one of the fusion partners has to be present on the target panel [22].

Although commercially hybrid-capture panels specific for RNA are available from Illumina (i.e., Trusight RNA fusion panel) and Agilent (i.e, SureSelect all-in One Solid tumor), few data have been published about their application on lung cancer specimens.

In 2019, Heyer and collaborators developed a targeted RNA sequencing (RNAseq) method that uses biotinylated oligonucleotide probes to enrich for RNA transcripts of interest with a double-capture approach (NimbleGen protocol—Roche, Basel, Switzerland), using an RNA input equal to or greater than 100 ng. This panel included 241 fusion genes and sequencing was performed on a HiSeq2500 Illumina instrument. After a validation phase, they tested 72 clinical samples across various tumors, including both fresh frozen and FFPE specimens and liquid samples (bone marrow and peripheral blood): the overall fusion gene diagnostic rate was improved from 63% with conventional approaches to 76%. Thirty-nine cases had already been reported as fusion positive by orthogonal methods: 33 out of 39 were confirmed positive by the NGS RNA panel; moreover, among 23 cases previously reported as negative, 12 were NGS positive. This could be due to the higher sensitivity of targeted RNAseq in comparison to traditional techniques or to a discrepancy between the isoforms detected by targeted RNAseq and those analyzed by FISH or RT-PCR. They evaluated also inter- and intra-run variability with a coefficient of variation equal to 0.073 and 0.071 respectively [44]. Always in this context, Kohsaka and collaborators developed a “junction capture method” in which capture probes are designed in a base interval of 120 bases from the putative junction sites. They considered that, according to the degree of fragmentation of nucleic acids from FFPE samples, sequence reads captured by the probes at distances of more than 120 bp from the junction points may not support fusion events. This panel showed a good performance in detecting 365 fusion genes as well as aberrantly spliced transcripts and the expression profile of 109 genes; and for what concerns gene fusions it was superior than genomic DNA testing [45].

DNA hybrid-capture panels are more common than the RNA ones in the setting of gene fusion analysis. Two DNA hybrid-capture panels are FDA-cleared and approved respectively: the Memorial Sloan Kettering (MSK) Integrated Mutation Profiling of Actionable Cancer Targets (IMPACT) and the FoundationOne CDx—Foundation Medicine (Roche). These panels allow the analysis of mutations, copy number alterations and structural rearrangements in 468 and 324 cancer associated genes, respectively, and both include the evaluation of microsatellite instability and tumor mutational burden. The MSK-IMPACT in comparison to FoundationOne Cdx requires the simultaneous analysis of tumor and normal DNA and both tests are executed only in specialized laboratories [46].

Other smaller DNA-targeted panels have been evaluated on FFPE clinical samples: a good concordance with gold standard methods and a good sensitivity and specificity was observed, the overall amount of DNA input required is usually equal to 50–250 ng [47,48,49,50,51,52]. Moreover, this approach has also been specifically tested on critical clinical samples like Endobronchial Ultrasound Guided Transbronchial Needle Aspiration (EBUS). Particularly, Xie and collaborators evaluated 85 FFPE EBUS specimens using the Lung Core 56 gene panel (Burning Rock Biotech; Asia-Pacific) and found that 77 samples also resulted as adequate when the percentage of tumor cells was as low as 5% [47]. Recently, Ruan et al. evaluated 108 malignant effusions from lung cancer patients; they used a panel including 17 lung cancer-associated genes and they successfully identified both gene mutations and rearrangements [48].

One of the drawbacks of methods based on hybridization capture is the relatively long workflow, so Pel et al. suggested a faster protocol called Linked Target Capture (LTC). This approach uses physically linked capture probes and PCR primers, it has been demonstrated that this technology can work on different sample types including ctDNA and FFPE-derived DNA, capturing a 35-gene pan-cancer panel, and detecting single nucleotide variants, copy number variants, insertions, deletions and gene fusions. With the integration of unique molecular identifiers (UMIs), they demonstrated that variants with as low as 0.25% abundance could be detected [21].

The analysis of gene fusions at DNA level offers important advantages: DNA is more stable than RNA and a unique NGS test can allow a complete tumor molecular characterization. Anyway, sensitivity of DNA-based NGS is not satisfying if fusion breakpoints involve long intronic regions that cannot be covered by hybridization-capture probes [9]. In fact, when compared with RNA-based analysis, DNA panel results are less accurate. Davies et al. compared FISH test, the RNA-based Archer Fusion Plex and a DNA hybrid-capture panel including 48 genes. Twenty cases were analyzed by FISH, 2 of which negative, but were reported as positive by both RNA and DNA NGS. Eighteen cases were analyzed by DNA sequencing: 4 were negative, but were reported as positive by FISH and RNA sequencing. Nineteen cases were analyzed by RNA NGS: 3 were negative, among these all were positive by FISH and 2 by DNA-NGS; all these negative samples presented a low-quality RNA. FISH misses a positive case, a *ROS1-GOPC* fusion; the two genes are closed within the same chromosome and this can explain why the break apart probe was not able to detect it; most DNA-NGS negative cases involve fusions within intron 31 of *ROS1*, which is characterized by repeated regions. Moreover, authors provided evidence that DNA and RNA breakpoints are not exactly the same, so at DNA level it is not always possible to predict gene fusion expression [53].

In the same way, Benayed and collaborators found that an RNA test can further improve tumor characterization in comparison to DNA sequencing. They selected 589 FFPE NSCLC cases that resulted in the wild-type and after an MSK-IMPACT test, 254 had enough material to purify RNA and to perform an analysis using an RNA panel based on Archer chemistry. They found that 33 had actionable alterations including gene fusions and *MET* exon 14 skipping, suggesting that, whenever possible, DNA sequencing for negative cases should be followed by RNA sequencing to increase the number of patients with targetable alterations. Some discordant cases might be due to the fact that the genomic breakpoint causing the rearrangement took place in an intron not tiled by the panel, in other cases the breakpoint involved again *ROS1* intron 31 full of repetitive regions [54].

### 2.4. Gene Fusions On Plasma Samples

In NSCLC tissue biopsies or FNA specimens are not always available and are barely representative of intra-tumor heterogeneity. Moreover, up to 10–20% of biopsies are inadequate for molecular testing [55,56]. Over the last few years, ctDNA has acquired a great relevance in the context of precision medicine of advanced lung ADC. It consists of short DNA fragments released in the circulation by tumor cells due to mechanisms like apoptosis or necrosis [55,57]. The use of ctDNA is approved to assess the presence of *EGFR*-activating mutations in advanced lung ADC patients whenever tumor tissue is not available, and to monitor EGFR TKI treatment [58]. In fact, patients presenting the *EGFR* T790M resistance mutation detected on ctDNA can be directly treated with the third generation EGFR TKI osimertinib. Considering that not all tumors shed sufficient amounts of DNA into the peripheral circulation, ctDNA analysis is informative only if the alteration is present also when the most sensitive assays are used [59].

Different NGS panels specific for ctDNA analysis have been developed and for what concerns gene fusions most of published data were obtained using hybrid-capture panels like Guardant 360 (G360) (Guardant Health, Redwood City, CA, USA) and FoundationOne Liquid (Foundation Medicine, Cambridge, MA, USA). G360 is a clinical panel specific for ctDNA which detects point mutations in up to 70 genes as well as copy number amplifications in 18 genes, fusions in six genes and small insertions or deletions in three genes. It is based on a paired-ends sequencing on Illumina platforms and requires a ctDNA input equal to 5–30 ng [60]. The FoundationOne Liquid panel allows an identification of base substitutions, insertions and deletions, copy number alterations and rearrangements in 70 commonly altered oncogenes. It is always based on a paired-ends sequencing system on Illumina platforms and requires 20–100 ng of input ctDNA [61].

One of the largest prospective studies was recently published by Leighl and collaborators; they evaluated the utility of G360 analysis in the identification of genomic biomarkers in treatment naïve NSCLC patients. A total of 282 patients were enrolled; tissue and plasma analyses identified respectively 60 and 77 patients with targetable alterations. In detail, 12 cases out of 60 positive tissue samples were negative on plasma, whereas 48 cases were positive on both tissue and ctDNA. For *EGFR*, *ALK*, *ROS1*, *B-Raf proto-oncogene*, *serine/threonine kinase (BRAF)*, concordance between ctDNA and tissue was greater than 98.2%, moreover ctDNA analysis increased the detection rate in wild-type tissue samples identifying more alterations [62]. McCoach and collaborators performed a survey of a laboratory cohort of *ALK*-positive lung cancer patients whit ctDNA tested by G360 assay to determine the clinical utility of plasma-based comprehensive genomic profiling. They have retrospectively identified 80 patients with 90 *ALK* fusions recorded on ctDNA: some cases were treatment naïve, other ones had insufficient tissue to perform FISH testing, other patients were on ALK TKIs progression and in some cases an *ALK* rearrangement was found in EGFR TKI resistant patients. It was proved that the use of ctDNA to complement tissue testing provided effective treatment options in these patients. Moreover, one case FISH negative and ctDNA positive for *ALK* fusion responded to an ALK TKI treatment [60].

Furthermore, Supplee and collaborators compared G360 panel with the ctDx-Lung panel (Resolution Bioscience, Redmond, Washington, USA), spanning exons and some introns of 20 NSCLC-associated genes, in 16 patients known to harbor an *ALK*, *ROS1* or *RET* fusion. G360 detected fusions in 7 cases, ctDx-Lung panel detected fusions in 13 cases, and 3 cases were detected by neither of them. The ctDX-Lung panel uses shorter capture probes in comparison to standard ones and another difference is the use of primer extension, which copies the sequence of the fusion partner regardless of the identity. It was demonstrated that differences in hybrid-capture techniques and bioinformatic calling methods are sources of variations in sensitivity among these assays, indeed by re-analyzing NGS data with updated software, more cases were identified also with the G360 panel [24].

For what concerns FoundationOne panel a prior version of the test, called FoundationACT (62 genes), was validated in the study by Clarck et al. In the validation phase on 2666 reference alterations this panel reached a high sensitivity and specificity also for gene fusion analysis with a minor allele frequency of 0.5%. It showed an inter- and intra-run reproducibility equal to 100%, no alterations were found in a group of healthy volunteers, concordance with tissue was equal to 75% and also a high concordance with orthogonal methods for ctDNA testing was reported. In addition, the results of prospective genomic profiling from 860 blood samples were assessed from patients with diverse cancer types and alterations were detected in ctDNA at comparable frequencies to tissue [61].

Wang et al. evaluated another capture-based targeted sequencing panel to detect and quantify rearrangement events. They used a commercially available panel (Burning Rock Biotech Ltd., Guangzhou, China) targeting 168 genes using 50 ng of ctDNA. Blood samples from 24 *ALK* rearranged cases, according to tissue biopsy testing, were analyzed: *ALK* rearrangements were successfully detected in 19 out of 24 patients at a baseline 100% specificity [63].

Plagnol et al. validated a panel based on an enhanced tagged amplicon sequencing which covers 36 genes for single nucleotide variations, indels, copy number variations and gene fusion events including key mutations in *EGFR* and *ALK* and *ROS1* fusions (InVisionFirst panel, Cambridge, UK). This panel proved to be able to detect gene fusions at high sensitivity, no differences were found between Streck and EDTA blood collection tubes and a high concordance was reported with digital droplet PCR, which is one of the election methods for ctDNA testing. They found that the use of short amplicons may result in a higher fraction of analyzed DNA in comparison to NGS methods including ligation and clean up. In this study authors reported a median depth of 69.000X, the target depth described for FoundationACT was greater than 5.000X unique median coverage [23] and the target depth for G360 v2.10 was about 15.000X [64].

Kunimasa tested an mPCR based target sequencing on an Ion PGM platform. They used a custom panel covering the entire *ALK* 19 intron frequently involved in rearrangements. They analyzed ctDNA from 20 *ALK* positive and 10 negative cases reporting a low sensitivity of 50% and a high specificity of 100%, using 10 ng of ctDNA [65].

Finally, some amplicon-based NGS panels analyze gene fusions at ctRNA levels together with gene alterations on ctDNA, like the commercially available Lung Cell Free Total Nucleic Acid kit (ThermoFisher). Papadopoulou et al. demonstrated that this approach is valuable in the molecular characterization of NSCLC patients. They analyzed 121 treatment naïve NSCLC plasma samples and 50 cases on EGFR TKI progression, reporting 49% of mutation-positive cases and a concordance with tissue equal to 86.11%. This assay allowed to characterize *ALK, ROS1* and *RET* fusions together with the most common genetic alterations by preparing a ctDNA and a ctRNA library. Moreover, such an assay uses random molecular tags as a unique molecular identifier to uniquely label each molecule prior to library amplification, thus increasing the test performance [66].

## 3. Discussion

Gene fusion analysis is crucial in the context of lung cancer precision medicine. Patients with tumors harboring *ALK*, *ROS1*, *RET* and *NTRK1/2/3* gene rearrangements can benefit from a treatment with tyrosine kinase inhibitors [67]. Although FISH and IHC are still the gold standard tests in most cases, NGS panels offer several advantages in the clinical practice. Gene fusions can be analyzed by NGS at DNA or RNA levels and in both cases targeted panels are preferred, they can be custom or commercially available, some of which approved for diagnostic use. Almost all panels analyzed in studies considered in this review showed a good agreement with conventional methods (FISH and IHC).

DNA-targeted panels for gene fusions are based on hybrid-capture methods and so on a gene-specific enrichment by a hybridization step with biotinylated DNA or RNA probes [21,44]. At DNA level it is possible to analyze gene fusion simultaneously to other gene alterations without purifying RNA and it allows to characterize both known and unknown fusion variants. On the other hand, diagnostic accuracy of DNA-based panels can be hampered by breakpoints within large intronic regions full of repetitive sequences and at DNA levels it is not possible to evaluate whether the fusion is or is not expressed [8]. It has been demonstrated that breakpoints at DNA and RNA levels are not always the same, because of post-transcriptional events and splicing modifications with important consequences on patient’s TKI sensitivity [68]. RNA-based approaches have the overall advantage of allowing an analysis of transcriptionally expressed gene fusions and sequencing is not affected by intronic regions [13]. In addition, RNA panels, besides gene fusions, allow a contemporary analysis of exon skipping events and expression levels of genes with a clinical value [42]. Although RNA analysis can be based on hybrid-capture or amplicon-based methods, most studies used the latter ones. Particularly, amplicon-based approaches can use a classical mPCR of an anchor mPCR. In the first case, only known fusion variants can be investigated, in the second case all the possible fusion variants of a gene included in the panel can be analyzed [38]. Moreover, amplicon-based methods on RNA allow the evaluation of the imbalance between the 3′ and 5′ regions of genes of interest, thus making it possible to also recognize gene fusions without knowing the exact partner and variant type [22]. RNA amplicon-based and, particularly anchored, mPCR assays proved to be highly sensitive and more accurate in the few available comparison studies with DNA gene fusion analysis [53,54]. However, this approach can suffer from PCR bias and requires a good quality RNA, which is sometimes difficult to be obtained from FFPE specimens. Benayed and collaborators in their study found that only 47% of cases had available tissue left for RNA extraction after conventional molecular characterization [54]. Although failure rate is acceptable across available studies, robust RNA extraction protocols are required, and it has been proved how the choice of extraction methods can impact on NGS analysis [69]. Moreover, biological material is often scarce, and it is not always possible to purify both DNA and RNA. In the studies evaluated in this review, different extraction methods have been used, both manual and automatized, in some cases RNA and DNA distinct kits were used [31,35]; in other works kits allowing to simultaneously separate DNA and RNA were preferred [29,30] and in some studies the use of total nucleic acids without splitting DNA from RNA was employed [42,43]. The possibility to use the same sections to purify both DNA and RNA can be advantageous, particularly for small biopsies and cytological specimens. In all cases, a quantitative and qualitative assessment of RNA is crucial, quantity is usually estimated by the Qubit fluorometer and quality assessed by Bioanalyzer testing or by specific quantitative PCR assays evaluating RNA amplifiability. RNA samples not fulfilling quality parameters can give false negative results [15,53]. However, today there is not an election method for nucleic acid purification for NGS analysis. Moreover, most data have been obtained on FFPE surgical and biopsy specimens and few data are available on RNA from cytological specimens, which are the only available material in about 50% of advanced lung ADC [17]. Cohen et al. reported a 30% dropout rate for cytological specimens and Haynes found that none out of 50 FNA smears had sufficient RNA to perform the analysis [39,42].

Nucleic acid purification is a crucial step also for liquid biopsies and it can be hampered by an inappropriate plasma separation, most studies directly use ctDNA [24,65], but in some cases gene fusion analysis requires ctRNA and specific purification procedures [66].

Besides nucleic acid extraction, NGS efficacy is greatly dependent also on library depth, the number of genes captured, the fusion gene expression levels and analysis pipelines. Most available kits are coupled with proper analysis systems and a number of total reads and fusion-specific reads to define a reliable test result is dependent on each panel and software. Notably, some NGS gene fusion tests have proved to be able to reveal low allele fractions with an excellent limit of detection and in some studies also samples with a low percentage of tumor cells had adequate results [31,40,41]. Another important technical aspect of gene fusion analysis by NGS methods is the possibility to characterize fusion variants. Currently, fusion variant determination is not required to treat patients with TKIs, but the more NGS is used, the more evidence is accumulating about different fusion variant TKI response [7,15,70].

As is widely discussed, NGS solutions for gene fusion analysis are different, in some cases samples are sent to specialized centers (MSK-impact, FuondationOne CDX, G360, Foundation One Liquid tests) and in other cases specific analytic workflows are established and validated within each laboratory, taking into account available expertise, the number of cases and, of course, costs. Indeed, NGS test reimbursement is still an open question in many countries. For instance, Cohen and collaborators suggested that sequentially combining DNA NGS and RNA NGS, executed only if DNA analysis does not reveal alterations, is the most cost-effective strategy for mutation and fusion detection in smoking-associated NSCLC cases, whereas for never-smokers they recommended a parallel approach [39]. Benayed proposed that all MSK-Impact negative cases should be tested by an RNA panel to better identify gene fusions and splicing alterations [54]. Turn-around time from sample arrival to report is another crucial point in the clinical practice: NGS analysis can require from 4 to 15 days and of course a sequential approach or test execution in specialized centers can require a few more days [28,39].

Furthermore, it has to be considered that NGS is not the only multiplex technique available for gene fusion analysis, in this context the NanoString nCounter system proved to be a valuable alternative [16]. It is based on a direct digital counting of mRNA molecules without any retro-transcription step; it allows to characterize only known fusion variants and to evaluate 3′ and 5′ gene regions imbalance. In comparison to NGS, it has a faster workflow and an easier data analysis pipeline. Moreover, it is less affected by RNA quality, because no retro-transcription or amplification steps are necessary. On the other hand, using NanoString, only known variants can be characterized and it generally requires a higher input RNA than some NGS panels [8,13]. Rogers and collaborators compared gene fusion analysis by three transcriptome-based platforms (NanoString, Agena LungFusion panel and ThermoFisher NGS fusion panel) to those obtained from *ALK, ROS1* and *RET* FISH. They analyzed 51 clinical specimens: NanoString showed an overall agreement of 96%, Agena of 94% and NGS of 86%; all platforms resulted highly sensitive [19]. No cases failed NGS analysis, whereas some cases failed NanoString testing. Vollbrecht’s group evaluated some FISH and IHC gene fusion concordant and discordant cases by both NanoString and NGS and found a great agreement between them [34]. There is not a perfect technique to analyze gene fusions but each one has its own pros and cons to evaluate according to the laboratory environment.

To sum up, in the context of gene fusion determination, NGS presents several advantages. First of all, it allows an analysis of more targets, saving time and material in an acceptable turn-around time. Then, some NGS approaches can reach high levels of sensitivity, also thanks to advanced analysis software, and have been proved to be adequate for different specimens: fresh frozen, FFPE biopsies and cell-blocks. Whereas, in spite of interesting available results, the real utility of NGS gene fusion testing on liquid biopsies in the clinical practice has yet to be better assessed on large-scale studies. Unfortunately, it is not easy to compare available studies because of different platforms, panels and protocols, study cohorts and analysis procedures; moreover, gene fusions are rare events and in some cases tests were conducted on a limited number of positive patients. Anyway, although a lack of standardization is evident, it is clear that a reliable NGS analysis stands on an appropriate validation procedure and protocols optimization, available interpretive software and bioinformatic support and on personnel expertise. Finally, NGS allows a constant incorporation of newly discovered biomarkers in the clinical practice and for gene fusion analysis, it has been acquiring a considerable relevance.

## Figures and Tables

**Table 1:** NGS Targeted Approaches for Gene Fusion Analysis.

	PROS	CONS
Hybrid-capture	Characterization of both known and unknown fusion variants of target genes Easily scalable to large gene panels Adequate for DNA and RNA gene fusion analysis At DNA level it does not require RNA purification and allows a simultaneous analysis of different gene variants	Higher RNA input than amplicon-based methods Difficulty with fusion variants involving large DNA intronic regions with repetitive sequences
Amplicon-based: ➢ Classical multiplex PCR (mPCR) ➢ Anchored multiplex PCR	Low RNA input Particularly effective with small and mid-size panels Analysis of both known and unknown fusion variants of target genes (anchored mPCR) 5′ and 3′ imbalance evaluation can increase test diagnostic accuracy	Not adequate for gene fusion analysis at DNA level Primer design can be complex Characterization of only known fusion variants included in the panel (classical mPCR) PCR bias like allele dropout can impact on analysis result
